# Pain and psychopathology after intensive care unit admission

**DOI:** 10.1177/0310057X241226716

**Published:** 2024-06-16

**Authors:** Nour Smaisim, Mienke Rijsdijk, Yuri van der Does, Arjen JC Slooter

**Affiliations:** 1 Pain Clinic, Department of Anaesthesiology, University Medical Centre Utrecht, Utrecht University, Utrecht, the Netherlands; 2 UMC Utrecht Brain Centre, University Medical Centre, Utrecht University, Utrecht, the Netherlands; 3 Department of Psychiatry, 8124 University Medical Centre Utrecht, Utrecht University, Utrecht, the Netherlands; 4 Department of Intensive Care Medicine, 8124 University Medical Centre Utrecht, Utrecht University, Utrecht, the Netherlands; 5 Department of Neurology, Universitair Ziekenhuis Brussel and Vrije Universiteit Brussel, Brussels, Belgium

**Keywords:** Intensive care unit, post-traumatic stress disorder, anxiety, depression, pain, psychopathology

## Abstract

Pain and psychopathology are observed in 18% and 55% of patients, respectively, 1 year after intensive care unit (ICU) admission. It is well known that chronic pain and psychopathology have a bidirectional relation in the general population, but it is not known whether this holds true for ICU survivors. The aim of this study was to investigate whether pain before, during and after ICU admission is related to psychopathology in ICU survivors 1 year after discharge. We performed a cohort study in a mixed ICU in the Netherlands between 2013 and 2016. At 1-year follow-up, patients completed the Hospital Anxiety and Depression Scale, the Impact of Event Scale/Impact of Event Scale-Revised, and answered standardised questions regarding pain. Psychopathology was defined as having anxiety, depressive and/or post-traumatic stress disorder symptoms. We used multivariable logistic regression analysis to evaluate the association of pain before, during and after ICU admission with psychopathology at 1 year follow-up. We included 1105 patients of whom 558 (50%) (95% confidence interval (CI) 0.48 to 0.54) had psychopathology at 1 year follow-up. Pain before ICU admission (odds ratio (OR) 1.18; 95% CI 1.10 to 1.26) and pain after ICU admission (OR 2.38; 95% CI 1.68 to 3.35) were associated with psychopathology. Pain during ICU stay was not associated with psychopathology, but the memory of insufficient pain management during ICU stay was (OR 2.19; 95% CI 1.39 to 3.45). Paying attention to pain and pain treatment experiences related to ICU admission may therefore contribute to early identification of ICU survivors at risk of psychopathology development.

## Introduction

Critical care survival has increased over the years.^1,2^ Quality of life, however, is often impaired in survivors,^3^ as intensive care unit (ICU) admission may expose patients to extreme physical and psychological stress, which may result in physical, mental and/or cognitive dysfunction. A third of ICU survivors appear to experience symptoms of anxiety or depression,^4,5^ and a fifth of patients report symptoms of post-traumatic stress disorder (PTSD) 1 year after ICU admission.^6^ More than half of ICU survivors (55%) show psychopathology 1 year after discharge.^7^ These numbers are comparable for various illness-related patient groups, such as survivors of acute respiratory distress syndrome or sepsis.^8^ Another frequent complication of critical illness is chronic pain,^9,10^ with an estimated incidence of 18% 1 year after ICU admission.^11^

Chronic pain has been related to anxiety, depression, and PTSD.^12,13^ The relationship between chronic pain and psychopathology in non-ICU patients appears to be bidirectional.^14^ Pain increases the risk of developing affective disorders, whereas psychopathology (anxiety, depression and distress) is a potent predictor for the transitioning from acute to chronic pain.^14,15^ Patients with chronic pain have an up to seven times higher incidence of experiencing symptoms of a mood disorder, compared to patients without chronic pain.^14^ It has been speculated that adequate pain control may prevent PTSD.^16^ However, it is not known whether pain before, during or after ICU admission is related to psychopathology development.

Therefore, in this study, we investigated whether pain before, during and/or after admission to the ICU was associated with psychopathological symptoms at 1 year follow-up.

## Methods

The procedures followed in this study were in accordance with the ethical standards of the Medical Research Ethics Committee of the University Medical Centre Utrecht and with the revised Helsinki Declaration of the World Medical Association (2013). The Medical Research Ethics Committee of the University Medical Centre Utrecht waived the need to obtain written informed consent (ethics approval no. 12/421: IC Delirium Database (16 October 2012), and ethics approval no. 10/006: Follow-up van IC patiënten een jaar na opname (12 January 2010)) as the data were routinely collected as part of an overall ICU quality control and improvement programme.

### Patient population

Data from adult patients who stayed for at least 48 h in a 32-bed, mixed medical-surgical ICU of the University Medical Centre Utrecht were prospectively collected between January 2013 and December 2016. Patients were excluded when they had been transferred from another ICU and therefore had incomplete data on their pain during their ICU stay. Patients with adverse neurological outcomes were also excluded as they are often not able to answer questionnaires. Patients who were alive 1 year after ICU discharge, according to the Municipal Personal Records Database, received a postal survey including the Hospital Anxiety and Depression Scale (HADS),^17^ the Impact of Event Scale (IES)/Impact of Event Scale-Revised (IES-R)^18–20^ and a structured interview on pain and memory of pain, including items from the Brief Pain Inventory (see supplemental material for a detailed description). The survey also included questions about the satisfaction of their ICU stay, physical complaints/complications after ICU admission, and whether they experienced limitations in their daily life and medication use. In order to increase the response rate, patients received a reminder questionnaire 1 month after non-response to the first survey. Non-responders received a telephone call 2 weeks after the reminder questionnaire was sent.

### Patient characteristics

Information on age, sex, admission type, Sequential Organ Failure Assessment (SOFA) score, Acute Physiology and Chronic Health Evaluation (APACHE) IV score, delirium during ICU stay (defined through a five-step algorithm^21^), C-reactive protein (CRP), and medication use before ICU admission was obtained from the electronic patient file. Medication use before ICU admission was subdivided into two groups: pain medication and psychotropic drugs. Pain medication included nonsteroidal anti-inflammatory drugs and/or opioids. Acetaminophen use was not reliably recorded and was therefore not assessed. Psychotropic drugs included neuropathic pain medication (amitriptyline, nortriptyline, duloxetine, gabapentin, pregabalin), antipsychotic drugs and/or antidepressants (all antidepressant drugs including amitriptyline, nortriptyline, and duloxetine). There was an overlap between neuropathic pain medication and antidepressants as some antidepressants are used as neuropathic pain medication.

### Pain

Information about pain before, during, and 1 year after ICU admission was collected with a standardised questionnaire (supplemental material) including the severity of pain and its impact on functioning,^22^ as follows.

Pain before ICU admission was assessed by asking participants at 1 year follow-up to identify body locations (maximum of 15) in which they experienced chronic pain before ICU admission. Pain before ICU admission was defined as one or more body part(s) with pain.

Pain during ICU admission was assessed three times daily by ICU nurses using a numeric rating scale (NRS) or the Critical Care Pain Observation Tool (CPOT). The NRS was the preferred tool and used whenever the patient was awake and could give an adequate response. In all other situations (e.g. sedation, delirious states) the CPOT was used. The NRS ratings vary from zero to 10, where zero indicates ‘no pain’ and 10 ‘the worst possible pain’. The CPOT involves four behavioural categories: facial expression, body movements, muscle tension, and ventilator compliance (for intubated patients) or vocalisation (for extubated patients).^23^ Pain during ICU admission was defined as having moderate to severe pain (NRS ≥4 or CPOT ≥2, yes/no) at least once during one ICU admission day and was incorporated in the models as the number of days with moderate to severe pain to include ‘time in pain’ in the variable.

We also assessed retrospective pain experience during ICU admission by asking about memories of pain during ICU stay at 1 year follow-up, (‘memories about pain during ICU admission, yes/no’) and whether participants experienced insufficient pain management (‘would have preferred more analgesics during ICU admission, yes/no’).

Onset of pain after ICU admission was assessed at the 1-year survey. Patients were additionally asked to score (0 to 10) the level of impairment due to pain in the last 24 h on each of the following domains: daily activities, mood, ability to walk, (domestic) work, social activities, sleep, and enjoying life. We defined impairment as the sum of the seven domains with a score between 0 and 70. Pain after ICU admission was defined as ‘new pain related to ICU admission, yes/no’.

### Psychopathology

Symptoms of anxiety and depression were assessed with the HADS.^17^ The anxiety and depression subscales of the HADS each consist of seven questions, asking about symptoms in the past 7 days. Patients scored each question on a four-point scale (0 to 3), with a maximum score of 21 per subscale. A score of eight or higher indicates clinically relevant anxiety or depressive symptoms.^17^

Symptoms of PTSD during the 7 days prior to questionnaire completion were measured either by the original IES with 15 items^18,19^ (sent to subjects admitted before September 2016) or the revised 22-item version^19^ (IES-R; sent to subjects admitted from September 2016 onwards (changed as part of the quality improvement programme)). Items in the original IES, representing subscales for intrusion and avoidance, were scored on a six-point scale: ‘not at all’ (0) to ‘often’ (5), giving a sum score ranging from 0 to 75. The IES-R includes seven additional items on hyperarousal. Items were scored on a five-point Likert scale: ‘not at all’ (0) to ‘extremely’ (4). Instead of raw sums, mean scores were calculated for the IES-R, ranging from 0 to 4. PTSD was defined as an IES sum score of 35 or more, or a mean IES-R score of 1.6 or more, indicating clinically significant PTSD symptoms.^19,20^

### Outcomes

Our primary outcome was psychopathology, defined as symptoms of anxiety (HADS ≥8), and/or depression (HADS ≥8), and/or PTSD (IES ≥35, or IES-R ≥1.6) 1 year after ICU admission. We also studied each symptom class separately.

### Model development

Prior to our analyses we deliberated extensively about the methodology.

The relationship between psychopathology and pain before, during and after ICU admission is complex, time-dependent and also influenced by a number of different factors. We considered using a mixed-model for statistical analysis but since the pain variables were based on different measures (number of pain locations before admission, NRS/CPOT scores during admission, and reported ICU-related pain after admission) in combination with the fact that pain before and pain after ICU admission was retrospectively measured (at 1 year follow-up) and pain during ICU admission prospectively (during the admission), we concluded that we could not use this model. Therefore, to prevent multicolinearity, we developed three separate multivariable logistic models with the outcome of psychopathology at 1-year follow-up and the variables pain before, during and after ICU admission. Covariables were a priori selected based on literature, clinical expertise and pathophysiologic reasoning.

The first model included pain before ICU admission and was adjusted for the following covariables: age, sex, psychotropic drug use before ICU admission (yes/no) and analgesic use before ICU admission (yes/no).

The second model included pain during ICU admission and was adjusted for the following covariables: age, sex, psychotropic drug use before ICU admission (yes/no), analgesic use before ICU admission (yes/no), type of admission (medical, acute surgical or elective surgical), cumulative SOFA score, APACHE IV score, delirium during ICU stay, and days with hyperinflammation (CRP ≥100 mg/L).

The third model included pain after ICU admission and was adjusted for the same covariables as in the second model.

### Statistical analyses

No statistical power calculation was conducted prior to the study. The sample size was based on all available data. We used multiple imputation to account for missing data.

Continuous data were expressed as mean with standard deviation (SD) or median with interquartile range (IQR), and categorical data as absolute numbers with percentages for the available data. The presence and severity of pain before, during and 1 year after ICU admission were compared between patients with and without psychopathology by an independent *t*-test for normally distributed data, a Mann–Whitney U test for non-normally distributed data or a chi-square test for categorical variables.

We calculated odds ratios (ORs) for pain before, during and after ICU admission and for the outcome of psychopathology using a multivariable logistic regression model containing the aforementioned covariates for confounding adjustment. We also evaluated the effect of pain measures on each psychopathology outcome separately by calculating the OR for symptoms of anxiety, depression, and PTSD. *P*-values less than 0.05 (two-sided) were considered statistically significant. Analyses were performed in SPSS version 25.0.0.2.

## Results

### Study population

**Figure 1.**
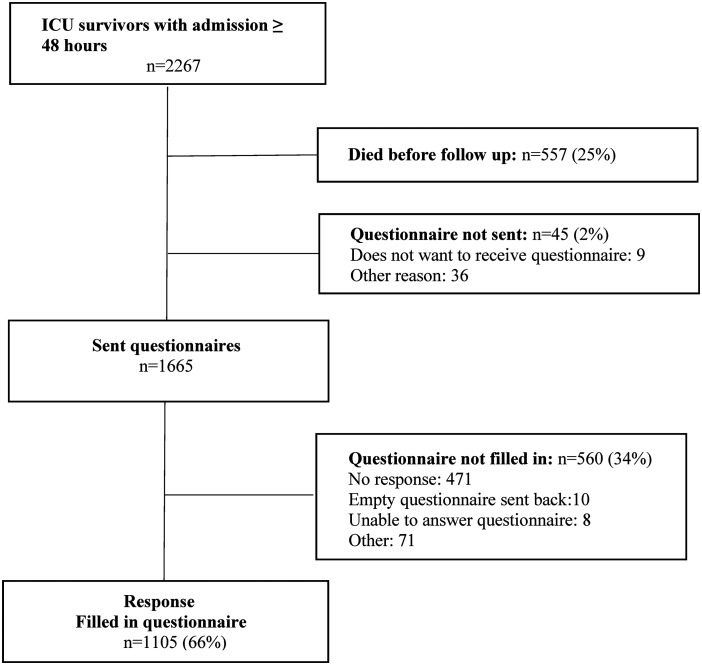
Study flowchart. Numbers represent patients in the dataset.

Of the 2267 eligible patients, 557 (25%) died before follow-up at 1 year. Of the 1710 survivors, 1665 received a questionnaire of whom 1105 (66%) responded (Figure 1). Patients admitted to the ICU were on average 59 years old (SD 16), 66.4% were male, and stayed a median of 5 days (IQR 3–10). See Table 1 for demographic and clinical characteristics.

**Table 1.** Demographic and clinical characteristics.

Demographic and clinical characteristics	Study population*N* = 1105
Age in years, mean (SD)	59 (16)
Male, *n* (%)	734 (66.4)
Type of ICU admission, *n* (%)	
Medical	463 (42.3)
Elective surgical	318 (29.0)
Acute surgical	314 (28.7)
APACHE IV score, mean (SD)	62.7 (24.6)
Cumulative SOFA score during ICU treatment, median (IQR)	84 (47–166)
Delirium during ICU stay,^a^ *n* (%)	620 (57.3)
Length of ICU stay, days, median (IQR)	5 (3–10)
Days with hyperinflammation (C-reactive protein >100 mg/L), mean (SD)	4.3 (5.5)

APACHE, Acute Physiology and Chronic Health Evaluation; ICU, intensive care unit; IQR, interquartile range; SD, standard deviation; SOFA, Sequential Organ Failure Assessment.

Missing data: Days with hyperinflammation (C-reactive protein ≥100 mg/L) 0.4%, cumulative SOFA score 0.5%, type of admission 0.9%, delirium during ICU stay 2.1%, APACHE IV score 20.4%.

a Defined through a five-step algorithm.^20^

Non-responders (*n* = 560) to the 1-year follow-up survey were younger (mean 53 years, SD 18, *p* ≤ 0.01), and were less often admitted after elective surgery (non-responders 21%, responders 29%, *p* < 0.01). There was no significant difference between responders and non-responders in APACHE IV score, cumulative SOFA score, or duration of ICU stay.

### Pain and psychopathology

**Figure 2.**
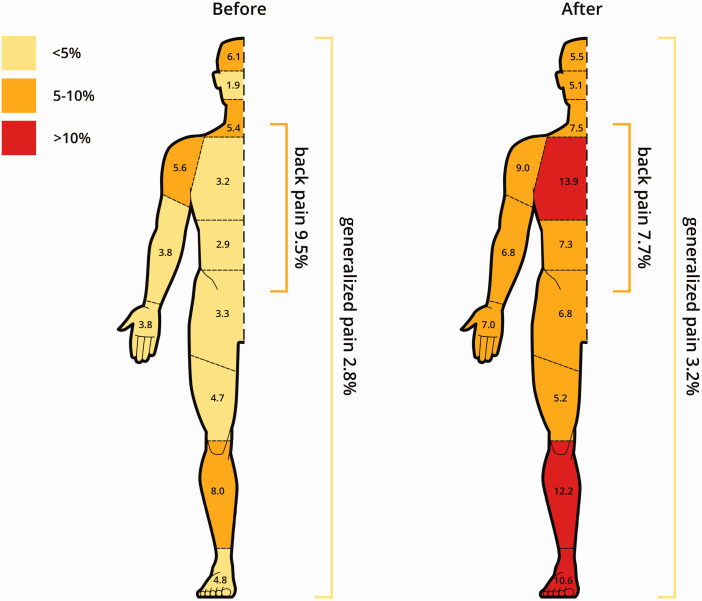
Affected body locations by pain before and 1 year after intensive care unit admission. Numbers represent percentages of patients of the total study population reporting pain in a specific body location. One patient can report pain in several locations. The colour coding represents frequency categories with yellow less than 5%, orange 5% to 10% and red more than 10% of patients reporting pain in this area.

Of the 1105 ICU survivors responding to the questionnaires, 558 (50%) reported symptoms of psychopathology at 1 year. A total of 372 (34%) participants indicated symptoms of anxiety, 370 (33%) reported depression-related symptoms, and 206 (19%) participants had developed symptoms of PTSD. Pain before ICU admission was reported by 338 (31%) participants, and by 172 (16%) participants at 1 year follow-up. Moderate pain (NRS ≥4 or a CPOT score ≥2) during ICU admission was reported by 825 participants (75%), severe pain (NRS >6) by 273 (27%). After-ICU-admission pain was most often located in the chest, lower leg, and feet (Figure 2). Pain and pain-related characteristics stratified by psychopathology at follow-up are presented in Table 2.

**Table 2.** Pain before, during and 1 year after intensive care unit treatment, stratified to psychiatric outcome at 1-year follow-up

Pain-related outcomes before, during and after ICU treatment	Psychopathology after 1 year*N* = 558	No psychopathology after 1 year*N* = 547	*P*-value
Pain before ICU			
Number of locations, median (IQR)	1.3 (0–2)	0.7 (0–1)	<0.001
at least one location, *n* (%)	212 (38.0)	153 (28.0)	<0.001
Pain during ICU			
NRS ≥4 or CPOT score ≥2, *n* (%)	422 (75.6)	403 (73.7)	0.750
Memories about pain during ICU admission, *n* (%)	168 (30.1)	159 (29.1)	0.538
Would have preferred more analgesics during ICU admission, *n* (%)	68 (12.2)	33 (6.0)	<0.001
Pain after ICU			
New pain related to ICU admission, *n* (%)	121 (21.7)	59 (10.8)	<0.001
Severity of pain (NRS 0–10), median (IQR)	2.9 (0–5)	1.5 (0–2)	<0.001
Moderate pain (NRS 4–6), *n* (%)	71(12.7)	37 (6.8)	<0.001
Severe pain (NRS ≥7), *n* (%)	72 (12.9)	22 (4.0)	<0.001
Impairment due to pain in last 24hrs, median (IQR)			
Daily activities	2.7 (0–5)	1.0 (0–1)	<0.001
Mood	2.3 (0–4)	0.7 (0–0)	<0.001
Ability to walk	2.9 (0–5)	1.2 (0–1)	<0.001
Work (domestic)	3.2 (0–6)	1.3 (0–2)	<0.001
Social contact	1.8 (0–3)	0.5 (0–0)	<0.001
Sleep	2.5 (0–5)	0.8 (0–1)	<0.001
Enjoying life	2.8 (0–5)	0.7 (0–0)	<0.001
Total impairment	14.0 (0–13)	0.0 (0–8)	<0.001
Medication use before ICU treatment, *n* (%)			
NSAID	153 (27.4)	141 (25.8)	0.221
Opioids	50 (9.0)	39 (7.1)	0.035
Neuropathic pain medication^a^	25 (4.5)	18 (3.3)	0.045
Antipsychotics	38 (6.8)	27 (4.9)	0.034
Antidepressants^b^	44 (7.9)	46 (8.4)	0.533

CPOT, Critical Pain Observation Tool; ICU, intensive care unit; IQR, interquartile range; NRS, numeric rating scale; NSAID, nonsteroidal anti-inflammatory drugs.

a Antineuropathic pain drugs include amitriptyline, nortriptyline, duloxetine, gabapentin and pregabalin.

b Antidepressants include all antidepressant drugs including amitriptyline, nortriptyline and duloxetine.

Pain before ICU admission was associated with an increased risk of psychopathology at 1 year follow-up (OR 1.18, 95% confidence interval (CI) 1.10 to 1.26) (Table 3). Pain during ICU admission was not associated with psychopathology development (OR 0.99; 95% CI 0.94 to 1.03). Pain after ICU admission was associated with psychopathology (OR 2.38, 95% CI 1.68 to 3.35). Results of the full models are presented as a supplemental material.

**Table 3.** Associations of pain before, during and 1 year after intensive care unit treatment with symptoms of anxiety, depression, post-traumatic stress disorder or psychopathology.

Pain and ICU treatment	PsychopathologyOR (95% CI)	Anxiety symptomsOR (95% CI)	Depressive symptomsOR (95% CI)	PTSD symptomsOR (95% CI)
Pain before ICU treatment^a^				
≥1 body part(s) with pain^a^	1.18 (1.10–1.26)*	1.14 (1.08–1.21)*	1.15 (1.08–1.22)*	1.10 (1.03–1.17)*
Pain during ICU treatment^b^				
Days with NRS ≥4 or CPOT score ≥2	0.99 (0.94–1.03)	1.01 (0.96–1.07)	0.93 (0.87–1.00)	1.05 (0.99–1.11)
Would have preferred more analgesics during ICU admission	2.19 (1.39–3.45)*	3.04 (1.96–4.71)*	2.13 (1.39–3.28)*	3.03 (1.92–4.77)*
Pain after ICU treatment^b^				
New pain related to ICU treatment	2.38 (1.68–3.35)*	2.49 (1.73–3.31)*	1.91 (1.36–2.67)*	2.79 (1.92–4.06)*
Impairment by pain after ICU treatment	1.06 (1.05–1.07)*	1.06 (1.04–1.07)*	1.06 (1.05–1.08)*	1.04 (1.03–1.05)*

APACHE, Acute Physiology and Chronic Health Evaluation; CI, confidence interval; CPOT, Critical Pain Observation Tool; ICU, intensive care unit; NRS, numerical rating scale; OR, odds ratio; SOFA, Sequential Organ Failure Assessment; PTSD, post-traumatic stress disorder.

a Adjusted for age, sex, psychotropic drug use before ICU admission drugs (antineuropathic pain drugs and/or antipsychotic drugs and/or antidepressants) and pain medication use before ICU admission (opioids and/or nonsteroidal anti-inflammatory drugs).

b Adjusted for age, sex, psychotropic drug use before ICU admission drugs (antineuropathic pain drugs and/or antipsychotic drugs and/or antidepressants), pain medication use before ICU admission (opioids and/or nonsteroidal anti-inflammatory drugs), type of admission, cumulative SOFA score, APACHE IV score, delirium during ICU stay (defined through five-step algorithm), days with hyperinflammation (C-reactive protein) ≥100 mg/L), duration of ICU stay (days).

Missing data: symptoms of depression/anxiety (HADS cut-off ≥8) 1.2%, psychopathology (HADS ≥8 or IES >35) 2.0%, days with pain, yes/no (NRS ≥4 or CPOT score ≥2) 1.8%, symptoms of PTSD (IES cut-off >35) 1.2%, ‘new pain related to ICU admission, yes/no’ 3.3%, ≥1 body part(s) with pain 4.5%, ‘would have preferred more analgesics during ICU admission, yes/no’ 6.1%, ‘total impairment by pain’ (0–70) 36.3%.

* *P*-value ≤0.05.

Insufficient pain management during ICU (‘would have preferred more analgesics during ICU admission, yes/no’: OR 2.19; 95% CI 1.39 to 3.45), and impairment by pain after ICU (‘impairment by pain’ (0–70): OR 1.06; 95% CI 1.05 to 1.07) measured at one year follow-up were both associated with psychopathology (Table 3).

**Figure 3.**
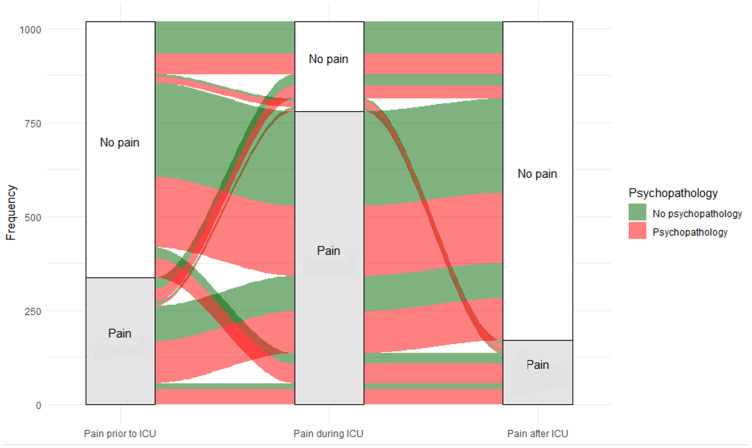
Alluvial diagram of patients with and without pain during follow-up. The boxes indicate the frequencies of patients who are diagnosed with psychopathology at 1 year follow-up. The red lines indicate the presence of psychopathology, the green lines indicate no psychopathology.

The pain trajectories of individual patients shown in Figure 3 indicate that there was substantial cross-over of participants. The majority of patients did not have pain before ICU admission, experienced pain during ICU admission, and had resolution of pain after ICU admission. The second largest group already had pain before ICU admission, which continued during ICU admission and resolved after ICU admission. The majority of patients with pain at 1 year also experienced psychopathological symptoms.

## Discussion

In summary, our findings suggest that at 1-year follow-up, half of former ICU patients had symptoms of psychopathology. Pain measured during ICU admission was not associated with psychopathology at 1-year follow-up, but the memory of insufficient pain management during ICU was. Pain before ICU admission and ICU-related pain at 1 year follow-up increased the odds of reporting psychopathology at 1-year follow-up.

The observed occurrence of psychopathology and pain in this study correlates with previous literature. Previous studies reported a prevalence of psychopathology at follow-up (3 to 60 months) between 52% and 55%.^7,23^ New-onset pain, in the first year after ICU discharge, was reported with cumulative incidences of 16% to 18%.^11,24^

The findings in our study correspond with the results of the Memory Study,^25^ in which patients recalled pain intensity and pain distress scores during ICU procedures that were significantly higher than reported during ICU admission. There is one study (*n* = 34) contradicting our findings, suggesting that pain measured during ICU admission (pain measured on the first day of admission) was associated with symptoms of PTSD in critically ill patients after mechanical ventilation.^26^ The study had a small sample size, and one could argue whether pain on the first admission day is a proxy for pain before or pain during ICU admission.

### ICU-related pain and psychopathology

The relation between pain and psychopathology has not been widely investigated in ICU survivors. However, extensive research has been performed in patients with chronic pain and psychopathology. These studies have shown that patients with conditions with pain have an increased risk of psychopathology.^27,28^ Although psychopathology is often referred to as a consequence of pain, prospective studies suggest that premorbid psychopathology increases the risk of developing chronic pain, making it a bidirectional relation.^14,28^ In our study we observed that the memory of insufficient pain management during ICU and impairment by pain after ICU were associated with psychopathology. It remains unclear which factors influence these experiences, and whether this may be a target of future preventive measures for psychopathology development.

### Strengths and limitations

This study is the first, to our knowledge, to investigate the effect of pain before, during and after ICU admission on psychopathology at 1 year follow-up. Our study was performed in a mixed ICU with a heterogeneous population, which increases the generalisability of the findings. We used multiple imputation to account for missing values. This strategy is widely regarded as superior to complete case analysis in mitigating biases.

However, the study had some limitations. First, approximately a third of the ICU survivors did not respond to our questionnaire. From the limited data we have, we know that these patients had comparable illness scores (no significant difference between responders and non-responders in APACHE IV score, cumulative SOFA score, or duration of ICU stay), suggesting that the non-responders were likely to have been in a comparable state at the time of follow-up. However, we cannot rule out that the non-response introduced some bias.

Second, newly acquired pain related to ICU admission was a self-reported outcome. Patients may have misclassified their pain as being ICU-related or have indicated their pre-existing pain as ‘newly acquired’, which could have led to either an over- or underestimation of newly acquired pain related to ICU admission. Patients with adverse neurological outcomes (e.g. traumatic brain injury) were excluded. As these patients often experience chronic pain,^29^ this may have resulted in a dilution of the observed effect. Furthermore, our survey unfortunately did not have questions to distinguish between nociceptive and neuropathic pain.

Another limitation is that there was no information available on psychopathology before ICU admission. We therefore used psychotropic drug use prior to ICU admission as a proxy measure for psychopathology. As a consequence, we might have underestimated the incidence of psychopathology before ICU admission since not everyone with psychopathology is medicated. Secondly, we were not able to make a distinction between psychotropic drug use at home or use started in the clinic/ward before ICU admission. This may have overestimated the chronic use of psychotropic drugs. In contrast to other studies, which used a telephone or face-to-face interview by a trained evaluator, a postal questionnaire/survey at 12 months was used to evaluate psychopathology after ICU admission. Patients with post-ICU-admission cognitive decline might have been unable to complete these surveys, and patients with depression might have been less willing to complete them. This could have introduced recall bias.

## Conclusion

Psychopathology at 1 year after ICU admission is prevalent, with approximately half of the patients reporting symptoms of anxiety, depression and/or PTSD. Pain measured during ICU admission was not associated with psychopathology at 1 year follow-up, but the memory of insufficient pain management during ICU admission was. Pain before ICU admission and new ICU-related pain were associated with a higher incidence of psychopathology.

Despite the limitations, this study is the first to assess the link between pain before, during and after ICU admission and psychopathology.

Assessment of pain before ICU and the monitoring of anxiety and depressive and PTSD symptoms after ICU discharge might contribute to early identification of patients at risk of developing psychopathology. Future studies are required to show whether early assessment of pain and in combination with analgesic interventions reduce the incidence of psychopathology.

## Supplemental Material

sj-pdf-1-aic-10.1177_0310057X241226716 - Supplemental material for Pain and psychopathology after intensive care unit admission Supplemental material, sj-pdf-1-aic-10.1177_0310057X241226716 for Pain and psychopathology after intensive care unit admission by Nour Smaisim, Mienke Rijsdijk and Yuri van der Does in Anaesthesia and Intensive Care
