# Supplementary material for: Pain and psychopathology after intensive care unit admission
Source: Anaesth Intensive Care. 2024 Jun 16;52(4):232–40. doi: 10.1177/0310057X241226716 (PMC11290044; doi:10.1177/0310057X241226716)
Supplement: sj-pdf-1-aic-10.1177_0310057X241226716 - Supplemental material for Pain and psychopathology after intensive care unit admission [file sj-pdf-1-aic-10.1177_0310057X241226716.pdf]

Supplementary files

Supplementary file 1. Pain questionnaire filled in at one year after ICU admission.

1.Do you experience NEW pain related to your Intensive Care admission?

Yes/no

2. Do you remember having pain during your Intensive Care admission?

Yes/no

3. Would you have wanted more analgesia during your Intensive Care admission?

Yes/no

4. Mark the areas of the body where you experience pain.

Pain can be present before Intensive Care admission or since Intensive Care admission.

Multiple boxes can be checked.

	Before ICU	After ICU
Pain in the lower body		
Pain in the		
Upper abdomen/ stomach		
Chest/ribs		
Headache and/or migraine		
Face, jaw or ears		
Neck		
Back		
Shoulders		
Arms/ elbows		
Hands		
Hip		
Legs, knees		
Feet		
Extensive part of the body /other pain		

5.The following questions concerning NEW pain related to ICU admission.

a. Please rate your pain by marking the number that describes your pain at its worst in the last 24 hours. 0 = no pain, 10 = worst pain.

0 1 2 3 4 5 6 7 8 9 10

b. Please rate you pain by marking the number that best describes your pain at its least in the last 24 hours . 0 = no pain, 10 = worst pain.

0 1 2 3 4 5 6 7 8 9 10

c. Please rate your pain by marking the number that best describes your pain on average.

0 = no pain, 10 = worst pain.

0 1 2 3 4 5 6 7 8 9 10

d. Please rate your pain by marking the number that best describes how much pain you have now. 0 = no pain, 10 = worst pain.

0 1 2 3 4 5 6 7 8 9 10

6. Mark the number that describes how , during the past 24 hours, pain has interfered your :

0 = does not interfere,10 = completely interferes.

A. General Activity

0 1 2 3 4 5 6 7 8 9 10

B. Mood

0 1 2 3 4 5 6 7 8 9 10

C. Walking ability

0 1 2 3 4 5 6 7 8 9 10

D. Normal work (includes both work outside the home and housework)

0 1 2 3 4 5 6 7 8 9 10

E. Relations with other people

0 1 2 3 4 5 6 7 8 9 10

F. Sleep

0 1 2 3 4 5 6 7 8 9 10

G. Enjoyment of life

0 1 2 3 4 5 6 7 8 9 10

Supplementary file 2. Table 1. Crude odds ratio’s

**Pain before ICU admission**				
Crude OR				
	Psychopathology	Anxiety symptoms	Depressive symptoms	PTSD symptoms
≥ 1 body part(s) with pain	1.18 (1.10-1.26)	1.14 (1.07-1.20)	1.15 (1.09-1.22)	1.11 (1.04-1.18)
Full model				
≥ 1 body part(s) with pain	1.18 (1.10-1.26)	1.14 (1.08-1.21)	1.15 (1.08-1.22)	1.10 (1.03-1.17)
Age, years	1.00 (0.99-1.00)	0.98 (0.98-0.99)	1.00 (1.00-1.01)	0.99 (0.98-1.00)
Sex, female	1.21 (0.93-1.57)	1.63 (1.25-2.14)	0.90 (0.68-1.18)	1.70 (1.23-2.34)
Psychotropic drug use before ICU admission[1]	1.16 (0.88-1.54)	1.09 (0.82-1.46)	1.11 (0.84-1.48)	1.07 (0.76-1.50)
Pain medication use before ICU admission[2]	1.14 (0.90-1.44)	1.03 (0.80-1.32)	1.13 (0.89-1.45)	1.31 (0.99-1.75)

[1] Use of neuropathic pain medication, antipsychotic drugs and/or antidepressants

[2] Opioids and/or nonsteroidal anti-inflammatory drugs

**Pain during ICU admission**				
Crude OR				
	Psychopathology	Anxiety symptoms	Depressive symptoms	PTSD symptoms
NRS ≥ 4 and/or CPOT ≥ 2	1.00 (0.97-1.03)	1.01 (0.98-1.05)	1.00 (0.97-1.03)	**1.06 (1.02-1.11)***
Would have preferred more analgesics during ICU admission	**2.11 (1.35-3.30)***	**3.19 (2.09-4.89)***	**1.91 (1.26-2.90)***	3.30 (2.14-5.08)*
Full model				
NRS ≥ 4 and/or CPOT ≥ 2	1.00 (0.97-1.03)	1.01 (0.98-1.05)	1.00 (0.97-1.03)	**1.06 (1.02-1.11)***
Would have preferred more analgesics during ICU admission	**2.11 (1.35-3.30)***	**3.19 (2.09-4.89)***	**1.91 (1.26-2.90)***	3.30 (2.14-5.08)*
Age, years	1.00 (0.99-1.01)	0.98 (0.98-0.99)	1.00 (1.00-1.01)	0.99 (0.98-1.00)
Sex, female	1.21 (0.93-1.57)	1.63 (1.25-2.14)	0.90 (0.68-1.18)	1.70 (1.23-2.34)
Psychotropic drug use before ICU admission[1]	1.16 (0.88-1.54)	1.09 (0.82-1.46)	1.11 (0.84-1.48)	1.07 (0.76-1.50)
Pain medication use before ICU admission[2]	1.14 (0.90-1.44)	1.03 (0.80-1.32)	1.13 (0.89-1.45)	1.31 (0.99-1.75)
Admission type<br>Medical	Reference	Reference	Reference	Reference
Surgical emergency	0.93 (0.70-1.25)	0.81 (0.60-1.10)	0.83 (0.62-1.12)	0.84 (0.59-1.20)
Surgical elective	**0.72 (0.53-0.96)***	**0.68 (0.50-0.93)***	**0.67 (0.49-0.91)***	**0.67 (0.45-0.97)***
Cumulative SOFA score	1.00 (1.00-1.00)	1.00 (1.00-1.00)	1.00 (1.00-1.00)	**1.00 (1.00-1.00)***
APACHE IV score	1.00 (1.00-1.01)	1.00 (0.99-1.00)	1.00 (1.00-1.011.66)	1.00 (0.99-1.00)
Delirium during ICU stay [3]	**1.44 (1.13-1.84)***	1.17 (0.90-1.51)	**1.66 (1.28-2.14)***	1.15 (0.84-1.56)
Days with hyperinflammation[4]	1.01 (0.99-1.04)	1.00 (0.98-1.03)	1.02 (0.99-1.04)	**1.03 (1.00-1.05)***
Duration of ICU stay, days	1.00 (0.99-1.01)	1.00 (0.99-1.01)	1.01 (0.99-1.02)	**1.02 (1.00-1.03)***

APACHE = Acute Physiology And Chronic Health Evaluation, CPOT= critical pain observation tool, ICU = intensive care unit, NRS= numeric rating scale, OR= odds ratio, SOFA = Sequential Organ Failure Assessment.

[1] Use of neuropathic pain medication, antipsychotic drugs and/or antidepressants

[2] Opioids and/or nonsteroidal anti-inflammatory drugs

[3] Defined through a five-step algorithm

[4] Days with C-reactive protein ≥ 100 mcg/L

*P-value < 0.05

**Pain after ICU admission**				
Crude OR				
	Psychopathology	Anxiety symptoms	Depressive symptoms	PTSD symptoms
New pain related to ICU admission	**1.18 (1.10-1.26)***	**1.14 (1.07-1.20)***	**1.15 (1.09-1.22)***	**1.11 (1.04-1.18)***
Impairment by pain after ICU admission	**1.06 (1.05-1.07)***	**1.05 (1.04-1.06)***	**1.06 (1.05-1.07)***	**1.04 (1.03-1.05)***
Full model				
New pain related to ICU admission	**1.18 (1.10-1.26)***	**1.14 (1.07-1.20)***	**1.15 (1.09-1.22)***	**1.11 (1.04-1.18)***
Impairment by pain after ICU admission	**1.06 (1.05-1.07)***	**1.05 (1.04-1.06)***	**1.06 (1.05-1.07)***	**1.04 (1.03-1.05)***
Age, years	1.00 (0.99-1.01)	0.98 (0.98-0.99)	1.00 (1.00-1.01)	0.99 (0.98-1.00)
Sex, female	1.21 (0.93-1.57)	1.63 (1.25-2.14)	0.90 (0.68-1.18)	1.70 (1.23-2.34)
Psychotropic drug use before ICU admission[1]	1.16 (0.88-1.54)	1.09 (0.82-1.46)	1.11 (0.84-1.48)	1.07 (0.76-1.50)
Pain medication use before ICU admission[2]	1.14 (0.90-1.44)	1.03 (0.80-1.32)	1.13 (0.89-1.45)	1.31 (0.99-1.75)
Admission type<br>Medical<br><br>Surgical emergency<br><br>Surgical elective	 Reference<br><br>0.93 (0.70-1.25)<br><br>**0.72 (0.53-0.96)***	 Reference<br><br>0.81 (0.60-1.10)<br>**0.68 (0.50-0.93)***	 Reference<br><br>0.83 (0.62-1.12)<br>**0.67 (0.49-0.91)***	 Reference<br><br>0.84 (0.59-1.20)<br>**0.67 (0.45-0.97)***
Cumulative SOFA score	1.00 (1.00-1.00)	1.00 (1.00-1.00)	1.00 (1.00-1.00)	**1.00 (1.00-1.00)***
APACHE IV score	1.00 (1.00-1.01)	1.00 (0.99-1.00)	1.00 (1.00-1.011.66)	1.00 (0.99-1.00)
Delirium during ICU stay [3]	**1.44 (1.13-1.84)***	1.17 (0.90-1.51)	**1.66 (1.28-2.14)***	1.15 (0.84-1.56)
Days with hyperinflammation[4]	1.01 (0.99-1.04)	1.00 (0.98-1.03)	1.02 (0.99-1.04)	**1.03 (1.00-1.05)***
Duration of ICU stay, days	1.00 (0.99-1.01)	1.00 (0.99-1.01)	1.01 (0.99-1.02)	**1.02 (1.00-1.03)***

APACHE = Acute Physiology And Chronic Health Evaluation, CPOT= critical pain observation tool, ICU = intensive care unit, NRS= numeric rating scale, OR = odds ratio, SOFA = Sequential Organ Failure Assessment.

[1] Use of neuropathic pain medication, antipsychotic drugs and/or antidepressants

[2] Opioids and/or nonsteroidal anti-inflammatory drugs

[3] Defined through a five-step algorithm

[4] Days with C-reactive protein ≥ 100 mcg/L

*P-value < 0.05
